# Draft Genome Sequence of a Xylanase-Producing Bacterial Strain, *Cellvibrio mixtus* J3-8

**DOI:** 10.1128/genomeA.01281-14

**Published:** 2014-12-11

**Authors:** Yi-Rui Wu, Bokun Lin, Yuli Yu

**Affiliations:** a Department of Civil and Environmental Engineering, National University of Singapore, Singapore; b School of Public Health, Guangdong Medical College, Dongguan, Guangdong, People’s Republic of China; c Department of Biology, Shantou University, Shantou, Guangdong, People’s Republic of China

## Abstract

The xylanase-producing bacterial strain *Cellvibrio mixtus* J3-8 was isolated from grassland giant snails. The draft genome of strain J3-8 comprises 5,171,890 bp in 152 contigs with a G+C content of 46.66%. This is the first genome report about this bacterial species.

## GENOME ANNOUNCEMENT

Recent research on the bioconversion from lignocellulosic materials into use in the biofuel industry has received increasing attention (1). Different from the costly and environmental unfriendly acid or heat pretreatment, enzymatic hydrolysis is considered to be one of the important and indispensable processes for bioconversion (2). Most bacteria have been reported as potential degraders of cellulose, dextran, xylan, chitin, and starch, including members of the *Cellvibrio* genus (2, 3). *Cellvibrio mixtus* J3-8 is a newly xylanase-producing soil bacterial strain isolated from grassland giant snails. The 16S rRNA gene of strain J3-8 shows 99.5% identity to that of *C. mixtus* subsp. *mixtus* strain ACM 2601 (GenBank accession no. AF448515), for which genomic data are not available. As *Cellvibrio* spp. have been considered the gene pool for degrading various polysaccharides (e.g., cellulose, dextran, chitin, and starch) (3, 4), here, we present a draft genome sequence of *C. mistus* J3-8.

The genome of *C. mixtus* J3-8 was sequenced by a whole-genome shotgun strategy using the high-throughput Illumina HiSeq 2000 at the Beijing Genomics Institute (Shenzhen, China). A total of 1,084,620 reads with a 500-bp insert size and totaling 542.31 Mbp were received, providing 105-fold coverage. The genome sequences were assembled *in silico* using SOAP*denovo* (version 1.05) (5), resulting in 152 contigs with an *N*_50_ length of 176,538 bp and a total length of 5,171,890 bp for the whole genome. These contigs were assembled into 50 scaffolds with a maximum fragment length of 1,327,211 bp. Putative protein-coding sequences were identified by Glimmer (version 3.02) (6) and analyzed by BLASTp. The functions of the predicted protein-coding genes were annotated via the databases Kyoto Encyclopedia of Genes and Genomes (KEGG) (7), Clusters of Orthologous Groups (COG) (8), UniProtKB/Swiss-Prot (9), and UniProtKB/TrEMBL (10). The identification of tRNAs and rRNAs was performed by tRNAscan-SE 1.21 (11) and rRNAmmer 1.2 (12), respectively.

The draft genome has 5,171,890 bases and a G+C content of 46.66%, and it contains 32 tRNA genes and 3 rRNAs (5S, 16S, and 23S rRNAs). Among 4,916 predicted genes (~88.82% of the total nucleotides), only 2,845 protein-coding sequences (CDSs) were identified (57.8% of the total obtained genes). A total of 2,030, 1,771, 825, and 478 proteins were annotated with the UniProtKB/TrEMBL, KEGG, COG, and UniProtKB/Swiss-Prot databases, respectively. The alignment analysis showed low identities for most genes, as 83.7% of them were lower than 90%.

In addition, there are a total of 145 CDSs involved in carbohydrate transport and metabolism, and 46 putative carbohydrate-hydrolase genes were detected in the whole genome, including those encoding cellulases, xylanases, glucanases, glucosidases, and xylosidases. The identities of these genes range only from 40.8% to 88.8%, which is significantly different from the submitted sequences in the GenBank database, even those in other *Cellvibrio* species. The relative poorly annotation percentage and the low identities of the genes indicate the uniqueness of this novel strain.

### Nucleotide sequence accession numbers.

The draft genome shotgun project has been deposited at DDBJ/EMBL/GenBank under the accession no. ALBT00000000. The version described in this paper is the first version, ALBT01000000.
